# Fluid Intelligence Is (Much) More than Working Memory Capacity: An Experimental Analysis

**DOI:** 10.3390/jintelligence11040070

**Published:** 2023-04-06

**Authors:** Dirk Hagemann, Max Ihmels, Nico Bast, Andreas B. Neubauer, Andrea Schankin, Anna-Lena Schubert

**Affiliations:** 1 Institute of Psychology, University of Heidelberg, 69117 Heidelberg, Germany; 2 Department of Psychology, University of Tübingen, 72076 Tübingen, Germany; 3 Department of Child and Adolescent Psychiatry, Psychosomatics and Psychotherapy, University Hospital Frankfurt, Goethe-University, 60590 Frankfurt am Main, Germany; 4 Department of Education and Human Development, DIPF Leibniz Institute for Research and Information in Education, 60323 Frankfurt am Main, Germany; 5 Institute of Business Psychology, FOM University of Applied Sciences, 45127 Essen, Germany; 6 TECO/Pervasive Computing Systems, Karlsruhe Institute of Technology, 76185 Karlsruhe, Germany; 7 Institute of Psychology, University of Mainz, 55122 Mainz, Germany

**Keywords:** working memory capacity, secondary tasks, fluid intelligence, working memory span tasks

## Abstract

Empirical evidence suggests a great positive association between measures of fluid intelligence and working memory capacity, which implied to some researchers that fluid intelligence is little more than working memory. Because this conclusion is mostly based on correlation analysis, a causal relationship between fluid intelligence and working memory has not yet been established. The aim of the present study was therefore to provide an experimental analysis of this relationship. In a first study, 60 participants worked on items of the Advanced Progressive Matrices (APM) while simultaneously engaging in one of four secondary tasks to load specific components of the working memory system. There was a diminishing effect of loading the central executive on the APM performance, which could explain 15% of the variance in the APM score. In a second study, we used the same experimental manipulations but replaced the dependent variable with complex working memory span tasks from three different domains. There was also a diminishing effect of the experimental manipulation on span task performance, which could now explain 40% of the variance. These findings suggest a causal effect of working memory functioning on fluid intelligence test performance, but they also imply that factors other than working memory functioning must contribute to fluid intelligence.

## 1. Introduction

There has been a long-lasting search for the particular cognitive processes that give rise to individual differences in intelligence. This quest has led to factors such as speed of information processing, attention, memory access, and transfer of information into the long-term memory, which have all served as candidates for a cognitive “core” of intelligence in terms of information processing (for reviews, see Mackintosh (2011) and Schweizer (2005)). In particular, working memory and individual differences in its capacity have repeatedly been considered as one important—if not the only essential—cognitive mechanism of general intelligence (Kyllonen 2002), fluid intelligence (Colom et al. 2015), or reasoning ability (Kyllonen and Christal 1990). This hypothesis has strong theoretical and empirical grounds.

Perhaps the most influential model of working memory was put forward by Baddeley and Hitch (1974; Baddeley 1986, 1997, 2007), who suggested that working memory comprises several components. A central executive component is involved in the control of processing and allocating attention resources, whereas two slave systems—the phonological loop and the visuospatial sketchpad—are engaged with temporary storage and rehearsal of speech-based and visuospatial-based information. According to Baddeley (1986), working memory is involved in all tasks that “require the simultaneous processing and storage of information” (p. 34).

In the five decades that followed the initial paper of Baddeley and Hitch (1974), there has been a proliferation of experimental paradigms and a plethora of empirical data. Some more contemporary examples for theories of working memory include the embedded processing model of working memory (Cowan 1999), the time-based resource-sharing model of working memory (Barrouillet et al. 2004), and the dual-component model of working memory (Unsworth and Engle 2007). After an extended survey of the empirical literature, Oberauer et al. (2018) suggested that there is no single theory of working memory that can explain all benchmark findings in this field of research. Still, most researchers would probably agree with the notion that “WM refers to a system or a set of processes, holding mental representations temporally available for use in thought and action” (Oberauer et al. (2018, p. 886), based on a survey of definitions by Cowan (2017)).

Regardless of the specific theoretic framework, it is safe to conclude that working memory must be involved in solving intelligence test problems that require reasoning and abstraction and thus necessitate the simultaneous processing and storage of information. Therefore, we would expect a substantial association between fluid intelligence and working memory capacity.

Moreover, a key feature of working memory is that its capacity is limited (Oberauer et al. 2018), although there is little agreement if time-based decay (Baddeley 1986; Barrouillet et al. 2004), interference between relational bindings (Farrell and Lewandowsky 2002; Oberauer 2019), or a cognitive resource (Popov and Reder 2020; Zhang and Luck 2008) limits its capacity. Individual differences in working memory capacity have been shown to reliably predict a broad variety of real-world tasks and ability measures such as reading comprehension, language comprehension, learning to spell, following directions, vocabulary learning, note taking, writing, and complex learning (see the review in Engle et al. (1999)). Because these abilities are typically related to fluid intelligence, there may be a tight empirical association between individual differences in fluid intelligence and the capacity limit of working memory. Further and more direct evidence stems from correlational research that included measures of intelligence and working memory capacity.

### 1.1. Correlational Studies of Reasoning Ability and Working Memory

Early evidence for a tight junction between intelligence and working memory—although not interpreted in these terms at the time—came from the work of Wechsler (1939), who developed the Wechsler–Bellevue Intelligence Scale (WBIS) as a new test battery for the measurement of intelligence. This test battery includes one subtest for the assessment of memory span, which was added to the battery due to its positive correlations with the other subtests. In later work, this subtest was labelled “digit span” and became part of the Wechsler Adult Intelligence Scale (WAIS; Wechsler (1955)). Further research has shown that digit span loads on a working memory factor and is nonetheless a fair measure of intelligence (for a review, see Zhu and Weiss (2005)). This test consists of two subtests. For the assessment of forward digit span, an experimenter reads out a string of numbers, and the participant has to repeat it. This subtest measures short-term storage capacity, which may correspond to the phonological loop in Baddeley’s (1986) model. For the measurement of backward digit span, the participant has to repeat the numbers in reverse order, thus this test imposes both storage and processing requirements and may match both the phonological loop and the central executive component. Jensen (1981) noted that backward digit span has almost twice the loading on intelligence compared to forward digit span, which he explained by the specific task requirements: “The main difference is that backward digit span requires more mental work and manipulation than forward digit span, which requires only reproductive memory” (p. 61). This observation suggests that it is rather the central executive component of the working memory system than the phonological loop which is essential for intelligence.

Some years later, Kyllonen and Christal (1990) reported in an influential paper on a very large association between working memory capacity and reasoning ability. Across four samples, they assessed reasoning with a variety of 15 tests, such as arithmetic reasoning, mathematical knowledge, AB grammatical reasoning, and verbal analogies. They further measured working memory capacity with six tasks, including ABCD grammatical reasoning, ABC numerical assignment, and digit span. A structural equation modelling (SEM) analysis of the data yielded correlations between the latent factors of working memory and reasoning in a range between 0.80 and 0.90 across the samples. The authors’ conclusion is presented in the title of their paper: “reasoning ability is (little more) than working-memory capacity” (p. 389).

Subsequent studies replicated and extended these results but usually reported a broader range of effect sizes of the relationship between working memory capacity and intelligence. For example, Engle et al. (1999) used operation span, reading span, and counting span tasks to measure working memory capacity, and the Standard Progressive Matrices (SPM; Raven et al. (1977b)) and the Culture Fair Test (CFT; Cattell (1973)) as measures of fluid intelligence. There was a correlation between the latent factors of working memory and reasoning in a size of 0.59. Most interestingly, Engle et al. (1999) also included measures of short-term memory into their study, which required only simple or no mental manipulations of the memorized items. They found that short-term memory did not explain variance in fluid intelligence beyond that explained by working memory capacity and thus concluded that the association between working memory capacity and fluid intelligence is driven by the central executive component.

There is a multitude of further correlational studies that measured reasoning ability and working memory capacity with a variety of tasks. Although these studies provided ample evidence for a positive correlation between both constructs, the magnitude of the resulting correlations shows considerable variability. Some meta-analyses aimed to integrate these findings. Ackerman et al. (2005) analysed the associations between working memory capacity and intelligence from 86 samples and found an average correlation of 0.48 between both constructs (CI = 0.44 to 0.52), which is substantially less than unity. Second, there is no gen. Kane et al. (2005) performed an independent meta-analysis where they only considered those 14 data sets that focused on fluid intelligence and that were analysed with a latent variables approach. They reported a correlation between latent factors of working memory capacity and fluid intelligence of 0.72 (range 0.41 to 1.00). Moreover, in a response to Ackerman et al. (2005), Oberauer et al. (2005) re-analysed the data by addressing some methodological shortcomings and reported a correlation between latent factors of working memory capacity and intelligence of 0.85.

Taken together, there is ample evidence for a substantial relationship between specific components of working memory and fluid intelligence, although the magnitude of the correlations shows variation between studies, which is likely due to methodological differences. On a latent variable level, the data suggest that there is about 50–60% of common variance shared by the two constructs. This finding has been interpreted as one of the “benchmarks” of working memory (Oberauer et al. 2018) or as support for a “quasi-isomorphic nature” of working memory capacity and fluid intelligence (Colom et al. 2015).

Before strong conclusions can be drawn from these data, some limitations must be noted. First, there was an overlap of item content of the tasks used to measure working memory capacity and reasoning ability in some of these studies (Lohman 2005; Süß et al. 2002). For example, Kyllonen and Christal (1990) used an arithmetic reasoning test (e.g., “Pat put in a total of 16 ½ h on a job during 5 days of the past week. How long is Pat’s average workday”) to measure reasoning ability in all four samples, and they used a mental arithmetic test (e.g., “8/4 = ?”) to measure working memory capacity in sample 1, 2, and 4, and a numerical assignment test (e.g., “A = B/2; B = C−4; C = 8; B = ?; A = ?; C = ?”) to measure the same construct in samples 1, 2, and 3. Individuals with good skills in arithmetic may do better in all of these tasks than individuals with poor arithmetic skills, thus the reported correlation between working memory capacity and intelligence may be partially due to individual differences in arithmetic skills.

Second, there is no general agreement on precise procedures or on mandatory test materials for the measurement of working memory capacity (for a thorough review of a variety of working memory span tasks and recommendations for their use, see Conway et al. (2005)). Because different studies used different measures, some differences in the size of the association between reasoning ability and working memory capacity may be due to methodological differences.

Third and most importantly, all studies reviewed so far are correlational and do not allow firm conclusions about the causal nature of the relationship between working memory capacity and fluid intelligence. For example, Kyllonen and Christal (1990) suggested that “working-memory capacity is responsible for differences in reasoning ability” (p. 427), although they also acknowledged that a reversed causal relationship may exist. More generally, Schweizer (2005) pointed out that the research into the cognitive basis of intelligence usually makes the presumption that simple mental activities explain complex mental activities, and thus properties of working memory may explain (cause) intelligence. Of course, it is challenging to test such a causal hypothesis with correlational methods, even if a sophisticated methodology of data analysis such as SEM is used (for a review of this methodological issue, see Shadish et al. (2002)). To make things worse, a correlation between two variables may be due to a third variable without any direct causal relationship between the two variables at hand. In particular, Baddeley (2007) suggested that there might be individual differences in motivation and effort while completing working memory capacity tasks and intelligence tests. For example, lack of motivation may lead some participants to put less effort into the working memory and reasoning tasks, and thus they do not bother to find strategies for a successful performance despite their potential of doing so. Taken together, the causal nature of the relationship between working memory functioning and fluid intelligence must be revealed with another approach.

### 1.2. Experimental Studies of Reasoning Ability and Working Memory

#### 1.2.1. Training Studies

Intervention studies provide a promising avenue for a causal analysis of working memory and intelligence. If differences in working memory capacity cause differences in intelligence, then an increase in working memory capacity by means of cognitive training should also improve intelligence.

In a pioneering study, Jaeggi et al. (2008) assigned participants to one of four experimental groups, which took part in 8, 12, 17, or 19 days of working memory training, or to respective passive control groups. The participants of the experimental groups trained working memory with a dual n-back task where the training program automatically adapted to the performance level of the user. The training took about 25 min each day. Most participants underwent a pre- and post-treatment test battery including a digit span task and reading span task for the measurement of working memory capacity and the Advanced Progressive Matrices (APM; Raven et al. (1977a)) or the Bochumer Matrizen Test (BOMAT; Hossiep et al. (1999)) for the measurement of fluid intelligence. The participants of the experimental groups showed an improvement in the dual n-back task across the training sessions. In addition, they also showed greater digit span (17% explained variance) and intelligence test performance (7% explained variance) at post-test in comparison to the passive control group. These gains in intelligence performance were moderated by the number of training sessions (7% explained variance). However, there was no training effect for the reading span task.

Although these findings are quite promising because they point to a causal effect of working memory functions on intelligence test performance, further studies failed to replicate this result. Chooi and Thompson (2012) assigned the participants in a well-controlled experimental study to one of six groups. The participants of two experimental groups trained working memory with an adaptive dual n-back task. The participants of two active control groups purportedly trained working memory with a dual n-back task with a fixed level of difficulty. There were additionally two passive control groups. In the experimental and the active control groups, participants trained once a day for 30 min for 4 days a week. Half of them trained for 8 days, and half of them trained for 20 days. In the passive control condition, participants waited for 8 days or 20 days. All participants underwent a pre- and post-treatment test battery including an operation span task as a measure of working memory capacity and the APM as a measure of fluid intelligence. The participants of the experimental groups showed an improvement in the dual n-back task performance of 34% after 8 days of training, and they showed an improvement of 44% after 20 days, respectively. In comparison to the control groups, there was, however, no transfer effect of any of the two training schemes on performance in the operation span task nor in the APM. Focusing on the experimental condition with 20 days of training, a descriptive pre-post-comparison shows a small increase in the operation span performance (6‰ of variance explained) but a very small *decrease* in APM performance (<1% of variance explained).

Redick et al. (2013) assigned the participants of a placebo-controlled experimental study to one of three groups. The participants of an experimental group trained working memory with an adaptive dual n-back task. The participants of an active control group worked on an adaptive visual search task, and there was a passive control group. In both training groups, there were 20 training sessions, which lasted between 30 and 40 min, and there was a limit of one session per day. The participants of both training groups underwent a pre-, mid-, and post-training test battery, and the participants of the passive control group were accordingly assessed. The test battery included a symmetry span and a running letter span task for the measurement of working memory capacity, the APM, seven other tests for the measurement of fluid intelligence, and a vocabulary and general knowledge test for assessment of crystallized intelligence. The participants of the experimental group showed an improvement in the dual n-back task performance across the 20 practice sessions (45% variance explained), and the participants of the active control group also showed an improvement in the visual search task performance (41% variance explained). A 3 (group) × 3 (pre-, mid-, post assessment) ANOVA of measures of working memory capacity or fluid or crystallized intelligence did not reveal any significant interaction effects. On a descriptive level, these interactions were of small size for the measures of working memory capacity (on average, 2% variance explained), fluid intelligence (on average, 3% variance explained), and crystallized intelligence (on average, 3% variance explained), respectively. Notably for the experimental group, there was a small pre-post increase in performance in both measures of working memory capacity, but there was a *decrease* in performance in six out of eight measures of fluid intelligence.

The findings of the latter two studies are consistent with the results of a meta-analysis by Melby-Lervac et al. (2016). They included 87 studies that investigated effects of working memory training on a variety of cognitive performance measures and reported three main findings from their analysis. First, there were large and significant effects of working memory training on performance in tasks that are highly similar or identical to those that have been trained (Hedges *g* for experimental vs. active control group: about *g* = 0.8, which corresponds to 14% explained variance). Second, there were moderate but significant transfer effects of training on measures of working memory capacity other than those that have been trained (about *g* = 0.3 and 2% explained variance). Third, there were no sizable effects of training on measures of nonverbal ability, verbal ability, decoding, reading comprehension, and arithmetic ability, which are more or less associated with intelligence (on average, *g* < 0.1 and explained variance < 1%). It is safe to conclude that the training of working memory (at least to the extent that has been realized in these studies) has no effect on performance in tests that measure intelligence or related constructs. Because fluid intelligence reflects a very broad and stable property of the neurocognitive system of an individual, it may not come as a surprise that a total training of 10 h (Chooi and Thompson 2012) or 12 h (Redick et al. 2013) with a specific working memory task is futile.

Other studies implemented more extensive training schemes that asked participants to not only train a single working memory task but a variety of experimental cognitive tasks. Some of these studies showed more promising preliminary results, see (Schmiedek et al. 2010, 2014; Steyvers and Schafer 2020), but others provided evidence that even extensive training of a variety of tasks related to working memory over five weeks has no sizable transfer effects (De Simoni and von Bastian 2018). Taken together, the working memory training approach has not succeeded in revealing a causal relationship between working memory and intelligence.

#### 1.2.2. Working Memory Load Studies

An alternative causal analysis of the relationship in question may be achieved with an experimental approach in which an intelligence test serves as the dependent variable, and load manipulations of the different working memory components serve as the independent variable. Baddeley and colleagues (Baddeley 1986, 1997, 2007; Baddeley et al. 1998; Baddeley and Hitch 1974; Logie et al. 1990) have developed and validated several procedures that may increase the load of specific components of working memory. These procedures can be used as a secondary task while the participants complete a standard intelligence test. If the causal hypothesis of Kyllonen and Christal (1990) holds and the hypothesis of Engle et al. (1999) is correct that only the central executive component is essential for fluid intelligence, then the intelligence test performance should be disrupted by a concurrent central executive task, but it should not be affected by concurrent phonological loop and visuospatial sketchpad tasks. A few studies manipulated the working memory load while the participants performed a reasoning task.

In some of their initial work, Baddeley and Hitch (1974, experiment 3) administered an AB grammatical reasoning task where the participants had to judge the correctness of sentences regarding the order of the two letters A and B. For example, the participant reads a sentence “A is not preceded by B—AB” in which case the correct answer is “true”. Concurrently to this task, the participants had to perform one of three working memory tasks. In a first condition, the participants were instructed to constantly repeat “The-The-The…”; in a second condition, they had constantly to repeat “One-Two-Three-Four-Five-Six”; and in a third condition, they had continuously to repeat a random six-digit sequence. There was a slowing down of reasoning time with increasing complexity of the material that had to be articulated (about 45% variance explained). This finding suggested that more complex materials capture more resources of the working memory system and leave less capacity to the reasoning processes, which may indicate a causal relationship of working memory on reasoning performance. One limitation of this study is that the manipulation of working memory aimed only at an articulatory suppression and thus does not allow separating the potential effects of the phonological loop, the visuospatial sketchpad, and the central executive. Moreover, it is not clear whether the AB grammatical reasoning task is a measure of intelligence or working memory capacity. For example, Kyllonen and Christal (1990, experiments 1 and 2) demonstrated that this task may serve equally well as a measure of both constructs. Thus, the finding of Baddeley and Hitch (1974) may alternatively suggest that a manipulation of the working memory load affects the performance of a working memory task, which may explain the great effect size but is a rather weak support for the hypothesized causal effect of working memory on intelligence.

Gilhooly et al. (1993, experiment 2) administered a syllogistic reasoning task which consisted of trials with two premises and asked for conclusions, i.e., “All A are B; All B are C; Therefore?”. Simultaneously, the participants had to perform one of three working memory tasks. In a first condition, the phonological loop was loaded with an articulatory suppression task that continuously demanded participants to repeat the numbers “1–2–3–4–5”. In a second condition, the visuospatial sketchpad was loaded with a task that asked the participants to press with their non-preferred hand four keys in a clockwise direction. In a third condition, the central executive was loaded by asking the participants to articulate the numbers 1–5 in a random order. In addition, there was a control condition without a secondary task. The response accuracy was smaller in the random number generation task than in the control condition (6% variance explained). Moreover, the reasoning time was longer in the random generation than in the control condition (15% variance explained). In contrast, neither the articulatory suppression task nor the key pressing task influenced reasoning performance. This finding suggests a causal effect of the central executive capacity on the performance of a reasoning task. However, these effects were too small in magnitude to allow a comprehensive explanation of intelligence in terms of working memory. This may be due to the choice of tasks in this study, because the syllogistic reasoning task is a rather specific measure of intelligence.

Klauer et al. (1997) administered propositional and spatial reasoning tasks, which required the participants to make a conclusion from premises that state the presence or absence of geometrical objects (e.g., “There is either a circle or a triangle; There is no triangle; ?”) or that state a spatial relationship between these objects (e.g., “The triangle is to the right of the circle; The square is to the left of the triangle; ?”). Concurrently, the participants had to perform several working memory tasks. In experiment 1, the central executive was loaded by asking the participants to articulate or type the numbers 1–9 in a random order. This resulted in a disruption of the response accuracy compared to a control condition (35% variance explained) and resulted in an increased reasoning time (38% variance explained). In experiment 2, the phonological loop was loaded with an articulatory suppression task that continuously demanded participants to repeat the numbers “1–2–3–4–5”. This manipulation also yielded a disruption of the response accuracy (8% variance explained) and an increase in reasoning time (77% variance explained). In experiment 4, the visuospatial sketchpad was loaded with a tapping task that required the participants to press the keys of a keypad in a set order, moving left–right and up–down over the keypad. This also resulted in a disruption of the response accuracy compared to a control condition (9% variance explained), but there was no effect on the thinking time. In principle, this study may have the same two limitations that were already noted for Gilhooly et al. (1993). Moreover, each of the three working memory components was loaded in a different experiment, which makes it somewhat difficult to compare the respective effects across the three components. In any case, it is puzzling that Klauer et al. (1997) found the greatest secondary task effects for an articulatory suppression condition, which is not consistent with the rest of the literature.

In a more recent study, Rao and Baddeley (2013) administered items of the SPM on printed pages and asked the participants to utter the number of the chosen response alternative, while participants underwent three concurrent tasks. In one condition, the central executive was loaded by a backward counting task. For this, a three-digit number was presented audibly (e.g., “seven-nine-three”) and the participant had to count down aloud in steps of two (e.g., “seven-nine-one, seven-eight-nine, …”). In another condition, the auditory loop was loaded by asking the participants to repeat the three-digit number continuously aloud. In addition, there was a silent baseline condition. There was a significant main effect of secondary task conditions on the error rate of SPM items (5% variance explained). However, post-hoc comparisons revealed no significant difference between the three conditions. In addition, there was a significant main effect of secondary task condition on the solution time of the SPM items (30% variance explained). The mean solution time was significantly longer in the counting backward condition than in the other two conditions, the latter of which did not show a significant difference. One limitation of this study is that the visuospatial sketchpad has not been loaded, which is somewhat surprising given the visual nature of any matrix reasoning test. Moreover, it is not clear that the secondary tasks were powerful enough to restrict the capacity of working memory severely and thus impair the quality of the SPM item solutions.

### 1.3. The Present Studies

Taken together, correlational studies showed large and robust associations between working memory capacity and fluid intelligence, which may approach a size of r = 0.70 to r = 0.80 (Oberauer et al. 2018). Nonetheless, this association may be partially due to an item content overlap (Ackerman et al. 2005). Moreover, conclusions are further limited because the typical study in this field of research uses complex span tasks for the measurement of working memory that may confound the capacity of different components or sub-processes of this memory system (Baddeley 2007). This problem may be solved with a latent variable modelling approach that allows us to decompose complex span tasks into latent sub-components (for fine examples, see the studies of Conway et al. (2004), Engle et al. (1999), and Kane et al. (2004)). Most importantly, however, the correlational approach does not allow conclusions regarding the causal nature of this association unless all relevant (and often unknown) confounding factors are controlled for (even in cases where the correlational data are analysed with a latent variable modelling approach, see Shadish et al. (2002, pp. 169–70)).

The latter problem may be solved with an experimental approach. Evidence from cognitive training studies suggests that the training in a working memory task may improve the performance in this task but has no sizable effect on measures of intelligence (Melby-Lervac et al. 2016). This may be due to the rather short training periods in most of these studies or the limited number of trained tasks that are not sufficient to change a stable property of the neurocognitive system, i.e., fluid intelligence.

A more direct approach for studying the experimental effects of working memory capacity on the performance in intelligence tests is the dual-task paradigm. The experimental work of Baddeley and Hitch (1974), Gilhooly et al. (1993), Klauer et al. (1997), and Rao and Baddeley (2013) provided unequivocal support for a causal relationship between the functioning of the working memory system and the performance in reasoning tasks. Nonetheless, these studies are not fully conclusive regarding the question of whether the capacity limit of working memory is the main cause of differences in intelligence. Limitations of these studies are an incomplete manipulation of the working memory system and—with the exception of Rao and Baddeley (2013)—a sub-optimal choice of the reasoning task as a measure of fluid intelligence. It must be noted that none of these studies’ aims was to test the causal relationship between working memory and intelligence, which is the very aim of the present study.

Bridging the fields of correlational and experimental disciplines (Cronbach 1957), we investigated how an experimental manipulation of working memory affected intelligence test performance. In study 1, participants completed items of the APM while they engaged in one of four secondary working memory tasks. In a first condition, the phonological loop was loaded with an articulatory suppression task that demanded participants to continuously repeat the numbers “1–2–3–4”. In a second condition, the visuospatial sketchpad was loaded with a task that asked the participants to press with their non-preferred hand four keys of a numerical pad in a clockwise direction. In a third condition, the central executive was loaded by asking the participants to articulate the numbers 0–9 in a random order. These tasks were used because they closely resemble the ones employed in previous research on working memory and reasoning (Gilhooly et al. 1993; Klauer et al. 1997) and because Baddeley and colleagues (Baddeley 1986, 1997, 2007; Baddeley et al. 1998; Baddeley and Hitch 1974; Logie et al. 1990) amassed evidence for the validity of these tasks. In particular, the magnitude of interference between the primary and secondary tasks indicates the degree of involvement of a working memory component in the primary task (Baddeley et al. 1984; Farmer et al. 1986; Gilhooly et al. 1993; Logie et al. 1989).

The APM was chosen as the dependent variable because factor-analytic research has demonstrated that this test has a maximum loading on fluid intelligence, nonmetric scaling studies have shown that it is at the core of the cognitive ability space, and because there is a general agreement in the literature that the APM may serve as a good proxy for fluid intelligence (Carroll 1993; Carpenter et al. 1990; Mackintosh 2011).

In study 2, we repeated the experiment of study 1 with the exception that we used three working memory tasks instead of the APM as the dependent variables. The purpose of study 2 was to test the validity of our working memory load manipulation and to estimate to what extent the performance in standard working memory tasks was impaired by the different memory load conditions in comparison to the impairments in APM performance in study 1. For this purpose, we used three established complex span tasks, namely the alphabet task (Kyllonen and Christal 1990), the computation span task (Ackerman et al. 2002), and the letter rotation task (Miyake et al. 2001).

The hypotheses of the present study are based on the presumption that individual differences in working memory capacity are an important cause of differences in fluid intelligence. We predicted that random number generation would load the central executive and thus disrupt APM performance as a measure of fluid intelligence. In contrast, we hypothesized that neither the articulatory suppression task nor the spatial key typing task would yield a disrupting effect because neither the articulatory loop nor the visuospatial sketchpad is crucially involved in intelligence. Moreover, we predicted that loading the central executive should affect intelligence test performance and working memory performance to a similar extent if working memory capacity is the main cause of individual differences in fluid intelligence. In this case, the APM and the working memory task would be isomorphic and thus measure the same thing across the experimental conditions.

## 2. Study 1

### 2.1. Materials and Methods

#### 2.1.1. Participants

The sample of this study consisted of 60 participants (13 male, 47 female; *M_age_* = 22.45, *SD_age_* = 4.7). All participants were students of the University of Heidelberg and received course credit for their participation in the study. Prior to the experiment, each participant was informed about the aim of the study and gave informed consent.

#### 2.1.2. Materials

*Dependent variable: Fluid intelligence.* Items of Set II of the APM were presented on a computer screen. In order to prevent fatigue and loss of motivation (particularly in the central executive condition), we created two test halves via an odd–even split of the 36 APM items. In the APM, the items are sorted by difficulty in ascending order. The use of an odd–even split preserved the item order in the two test halves. Each item consisted of a 3 × 3 matrix with one missing segment. The participants were instructed to complete the matrix by choosing from 8 alternatives which were numbered from 1 to 8. The participants scored 1 point for each correct solution. Across both test halves and all experimental conditions, the mean score was *M* = 8.93 (*SD* = 3.32), and Cronbach’s alpha was α = 0.70.

*Working memory span.* Working memory span was tentatively assessed with the digit forward–backward task from the WAIS (Wechsler 1955). Each correctly repeated sequence was scored with one point. The mean score in our sample was *M* = 17.25 (*SD* = 4.28) for the whole task, with *M* = 8.88 (*SD* = 2.09) for the digit forward task and *M* = 8.37 (*SD* = 2.76) for the digit backward task. The correlation between the two test halves was *r* = 0.55.

*Fluid Intelligence.* Participants’ intelligence was measured with the German form of the Culture Fair Test 3 (CFT 3; Cattell and Weiß (1971)). For each of the four parts of the test, participants had 2.5 to 4 min of time. The mean overall score in our sample was *M* = 28.48 (*SD* = 4.49), which corresponds closely to the German standard norms of university students (*M* = 25.7 and *SD* = 4.0; Cattell and Weiß (1971)).

#### 2.1.3. Procedure

At the beginning of the experiment, participants completed the digit forward–backward task from the WAIS, followed by the CFT 3. After a short break, they worked on a computerized version of the APM while performing a secondary task. Each APM item was presented together with a set of possible solutions including the correct solution. As soon as the participants were ready to answer, they had to press the spacebar and then enter the number of their solution on a regular keyboard with their dominant hand. After first completing a practice item taken from the Standard Progressive Matrices (SPM; Raven et al. (1977b)), participants had to solve 18 APM items in 3 blocks of 6 items each with a short break between each block. For each item, accuracy and answering time were recorded. The number of correct item solutions (APM score) and the total reasoning time for the correctly solved items (APM reasoning time) served as dependent variables in statistical analyses.

Before the computer-based APM started, participants were given written instructions about their secondary task. Each participant had to perform one of four secondary tasks while solving the APM: generating random numbers (loading the central executive), repeating a sequence of numbers (loading the phonological loop), pressing a sequence of keys (loading the visuospatial sketchpad), or performing no secondary task (control group). While working on the APM, participants in all conditions listened to the beat of a metronome at a rate of 60 beats per minute.

*Random number generation.* Participants were instructed to generate a random sequence of numbers using the numbers 0 to 9. To explain the principle of random sampling with replacement, we asked participants to imagine they pull a ball out of an urn, read out the number written on the ball, then put the ball back into the urn, shuffle all balls, and then draw a new one, etc. Participants were instructed to articulate one number immediately after each beat of the metronome.

*Key pressing.* Participants were instructed to repeatedly press four keys of a separate numerical pad in a clockwise manner with their non-dominant hand. Using a separate numerical pad ensured that the participants were able to tap the rhythm comfortably. Unused keys were masked by carton to prevent the participants from key slipping and to help them focus their gaze on the computer screen. They had to press one key immediately after each beat of the metronome.

*Counting task.* The Participants were instructed to count from 1 to 4 and then start anew at 1. They had to articulate one number immediately after each beat of the metronome.

*Control task.* Participants were made aware of the metronome and instructed to ignore it.

After the participants had finished working on the APM we recorded age and sex.

#### 2.1.4. Design and Data Analysis

Participants were randomly assigned to one of the four experimental conditions (control group, phonological loop, visuospatial sketch pad, and central executive). The mean structure of APM score and APM reasoning time was investigated with an analysis of variance (ANOVA), followed by planned comparisons. All experimental effects were quantified with Hays (1994) ω^2^, which is a partial effect size that estimates the proportion of explained variance in between-subject designs.

To check for pre-experimental differences in working memory capacity and fluid intelligence between the four experimental groups, we measured participants’ working memory capacity with the digit forward–backward task and their intelligence with the CFT. Across the four experimental groups, there were no systematic differences in participants’ working memory capacity (*F*(3, 56) = 1.42, *p* = .247, ω^2^ = 0.02) nor their fluid intelligence (*F*(3, 56) = 0.90, *p* = .447, ω^2^ = 0.00). This indicates that the randomization of participant assignments to experimental groups was successful regarding the constructs of interest.

The type I error probability was set to α = 0.05. With a given sample-size of *N* = 60, an F-test of the secondary task factor has a statistical power of 1 − *β* = 0.93 if the population effect size is *f* = 0.50 (Erdfelder et al. 1996), thus meeting the criterion proposed by Cohen (1988) for the interpretation of the null hypothesis.

### 2.2. Results

#### 2.2.1. APM Scores

**Figure 1:**
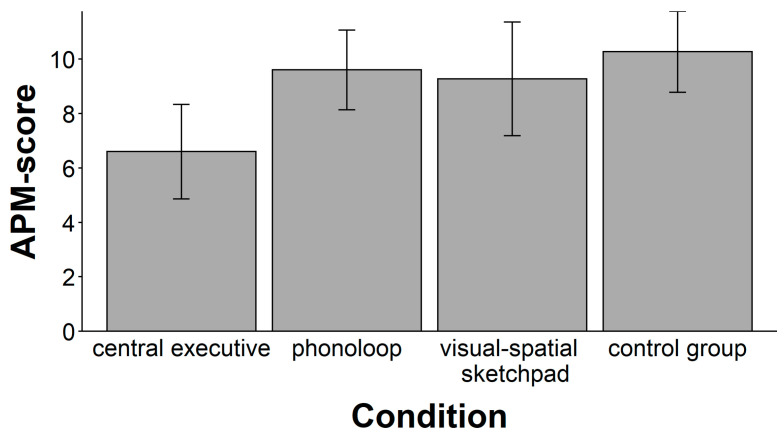
Mean APM performance in the four different groups. Whiskers indicate 95% confidence intervals.

There was a significant main effect of the secondary task (see Figure 1), *F*(3, 56) = 4.07, *p* = .011, ω^2^ = 0.13. A follow-up with planned comparisons showed that the mean performance in the central executive condition was significantly lower than the mean performance in the other groups, *t*(58) = 3.41, *p* < .001, ω^2^ = 0.15. In contrast, the mean performance did not differ between the other three groups, *F*(2, 42) < 1.

#### 2.2.2. APM Reasoning Time

There was no significant main effect of the secondary task on APM reasoning time, *F*(3, 56) = 0.89, *p* = .452, ω^2^ = 0.00.

### 2.3. Discussion

The main result of study 1 is that an experimental manipulation of working memory load affected performance in the APM. Loading the central executive with a secondary task diminished the number of correctly solved APM items, whereas loading the phonological loop or the visuospatial sketchpad had no deteriorating effect. This finding is consistent with the proposal that working memory capacity is intrinsically related to fluid intelligence (Bühner et al. 2005; Colom et al. 2004; Engle et al. 1999; Kyllonen and Christal 1990). Moreover, this finding renders further support for the proposition that it is the central executive rather than the two slave systems that sustains the performance in intelligence test (Engle 2002). Compared to the results of Rao and Baddeley (2013), there are many similarities and a few differences. We also found that only loading the central executive had a sizable effect on APM performance, whereas loading the slave systems did not impair APM performance. In contrast to their study, however, we found a medium effect size of dual tasks on the APM test score that explained 15% of variance, whereas Rao and Baddeley (2013) reported that only 5% of the variance in SPM test scores could be explained. Conversely, we found no effect of dual tasks on the APM reasoning time, whereas Rao and Baddeley (2013) reported that 30% of the variance in SPM reasoning time could be explained. It is important to note that participants in both studies were instructed to respond as accurately as possible rather than fast, as it is the standard practice in administering the SPM and APM, respectively. Hence, the differences in results may be due to the use of the different dual tasks or the different matrix tests.

In any case, it should be noted that our experimental manipulation of working memory explained only 15 % of the variance in fluid intelligence test performance. This magnitude is clearly at odds with proposals claiming a quasi-isomorphic nature of both constructs or that working memory is the most important mechanism of fluid intelligence, which has been suggested by correlational research. Instead, this result suggests that working memory capacity may be only one of several factors contributing to individual differences in fluid intelligence.

One objection to this conclusion might be that it is only valid under the presumption that the secondary tasks of the present study yielded a sufficient working memory load. For example, if the central executive task of the present study loads only about 10–20% of working memory capacity, then 80–90% of its capacity remains free for working on intelligence test items. Accordingly, the experimental effects would be rather small. Although this objection is theoretically sound, there is some contrary evidence reported in the literature. In a comparable study, for example, Baddeley and Hitch (1974, experiment 3) manipulated working memory load with secondary tasks as an independent variable and used reasoning time in an AB grammatical reasoning task as the dependent measure, the latter of which may capture working memory capacity and intelligence equally well, see (Kyllonen and Christal 1990, experiments 1 and 2). In this study, the experimental manipulation could explain about 40% of the variance in reasoning time, which suggests that our secondary tasks should yield a sufficient working memory load.

To estimate how much variance in working memory can really be explained by our experimental manipulation, we conducted a second study in which we assessed the effects of the secondary tasks on working memory capacity instead of intelligence, as in (Baddeley and Hitch 1974). Because working memory tasks employ very specific contents (e.g., verbal, numerical, or spatial material), we used the performance in three working memory tasks tapping these different domains as dependent variables. We hypothesize that replacing the dependent variable with a task measuring working memory should substantially increase the experimental effects of the secondary tasks and should particularly increase the effect of the random number generation condition. Such a result (i.e., observing a much larger effect of random number generation on working memory capacity than intelligence) would be difficult to explain under the presumption that working memory capacity and fluid intelligence were the same.

## 3. Study 2

### 3.1. Materials and Methods

#### 3.1.1. Participants

The sample of this study consisted of 60 participants (16 male, 43 female; *M_age_* = 21.24, *SD_age_* = 2.2). One participant’s data was lost due to equipment failure and another participant’s performance in the alphabet task could not be saved due to a system crash during the task. All participants were students of the University of Heidelberg and received course credit for their participation in the study. Prior to the experiment, each participant was informed about the aim of the study and gave informed consent.

#### 3.1.2. Material

*Dependent variables: Working memory tasks.* Working memory was measured by the following three tasks using different content (verbal/numerical/spatial).

*Alphabet Task.* In the alphabet task (Kyllonen and Christal 1990), participants saw a string with a varying number of letters for 3 s on a computer screen. They then had to apply successor and predecessor operations to the string of letters. If the string presented on the first screen, for example, consisted of the letters A, L, C and the operation on the second screen was +1, the correct response was B, M, D. Participants either had to add or subtract 1 or 2 to the string of letters. These operations were displayed for 1.5 s and participants had unlimited time to respond. The difficulty increased over trials from three to seven letters (5 levels × 4 trials = 20 trials total). The number of correct trials was used as the dependent variable. Across all experimental conditions, the mean score was *M* = 5.45 (*SD* = 4.44), and Cronbach’s alpha was α = 0.87.

*Computation Span.* In the computation span task (Ackerman et al. 2002), participants saw mathematical equations and had to decide whether the displayed solution of the equations was correct. Moreover, they had to memorize the solution irrespective of its accuracy. After a number of equations ranging from three to seven, they had to reproduce the displayed solutions in sequential order. Equations were presented on a computer screen for 6 s and participants had unlimited time to respond. Moreover, they also had no time limit when recalling the solutions. Difficulty increased over trials from three to seven equations (5 levels × 3 trials = 15 trials total). The number of correctly recalled solutions was used as the dependent variable. Across all experimental conditions, the mean score was *M* = 5.66 (*SD* = 5.06), and Cronbach’s alpha was α = 0.93.

*Letter Rotation.* In the letter rotation task (Miyake et al. 2001), participants saw a series of pictures of capital letters (F, J, L, P, or R). Each letter appeared mirror-imaged or normal and in one of seven possible rotations (multiples of 45°, except the upright orientation) for 3 s. Participants then had to indicate whether the letter was mirror imaged or normal using two hotkeys on the keyboard. Additionally, they had to remember the spatial orientation of the letter. After of a certain number of letters, they were asked to indicate the positions of the tops of the formerly presented letters in the correct order. This could be done by using the numerical pad of the computer keyboard (7 representing top-left, 4 representing left, etc.). Trial difficulty increased from two to five letters (4 levels × 3 trials = 12 trials total). The number of correct trials was used as the dependent variable. Across all experimental conditions, the mean score was *M* = 4.86 (*SD* = 3.62), and Cronbach’s alpha was α = 0.85.

In addition, working memory span and fluid intelligence were assessed with the following tasks.

*Working memory span.* Working memory span was tentatively assessed with the digit forward–backward task from the WAIS (Wechsler 1955). Each correctly repeated sequence was scored with one point. The mean score in our sample was *M* = 17.86 (SD = 3.68) for the whole task, with *M* = 9.56 (*SD* = 2.05) for the digit forward task and *M* = 8.31 (*SD* = 2.44) for the digit backward task. The correlation between the two test halves was *r* = 0.34

*Fluid Intelligence.* Participants’ intelligence was measured with the German form of the Culture Fair Test 3 (CFT 3; Cattell and Weiß (1971)). The mean overall score in our sample was *M* = 30.34 (*SD* = 4.18), which is higher than the German standard norms of university students (*M* = 25.7 and *SD* = 4.0; Cattell and Weiß (1971)).

#### 3.1.3. Procedure

The procedure of study 2 was similar to the procedure of study 1. After completing the CFT and the digit forward–backward task, participants started with their assigned secondary task (random number generation, key pressing, counting, or control condition). Instructions for this task were given in written form. As in study 1, each participant had to perform one of four memory load tasks. After starting with the secondary task, participants completed the three working memory tasks described above (alphabet task, computation span, and letter rotation) instead of the APM of study 1. The order of tasks was balanced across participants with short breaks between each task. Participants in all conditions listened to the beat of a metronome with a rate of 60 beats per minute while completing the working memory tasks.

#### 3.1.4. Design and Analysis

Participants were randomly assigned to one of the experimental conditions (control group, phonological loop, visuospatial sketch pad, and central executive). We analysed the dependent variables (alphabet task score, computation span, and letter rotation score) with a multivariate analysis of variance (MANOVA) and three separate analyses of variance (ANOVAs), followed by planned comparisons. All experimental effects were quantified with Hays (1994) ω^2^, which is a partial effect size that estimates the proportion of explained variance in between-subject designs.

To check for pre-experimental differences in working memory capacity and fluid intelligence between the four experimental groups, we measured participants’ working memory capacity with the digit forward–backward task and their intelligence with the CFT. Across the four experimental groups, there were no systematic differences in participants’ working memory capacity (*F*(3, 55) = 0.93, *p* = .431, ω^2^ = 0.00) nor their fluid intelligence (*F*(3, 55) = 0.98, *p* = .409, ω^2^ = 0.00). This indicates that the randomization of participant assignments to experimental groups was successful regarding the constructs of interest.

The type I error probability was set to α = 0.05. With a given sample-size of *N* = 60, an *F*-test of the secondary task factor has a statistical power of 1 − *β* = 0.93 if the population effect size is *f* = 0.50 (Erdfelder et al. 1996), thus meeting the criterion proposed by Cohen (1988) for the interpretation of the null hypothesis.

### 3.2. Results

#### 3.2.1. General Memory Load Effects

We computed a MANOVA with the three working memory tasks as dependent variables and found a significant main effect for the secondary task, *F*(9, 127) = 7.21, *p* < .001, ω^2^ = 0.24, Wilk’s Λ = 0.37. We then computed separate ANOVAs for each of the three dependent variables to test for specific memory load effects on the working memory tasks.

#### 3.2.2. Alphabet Task

**Figure 2:**
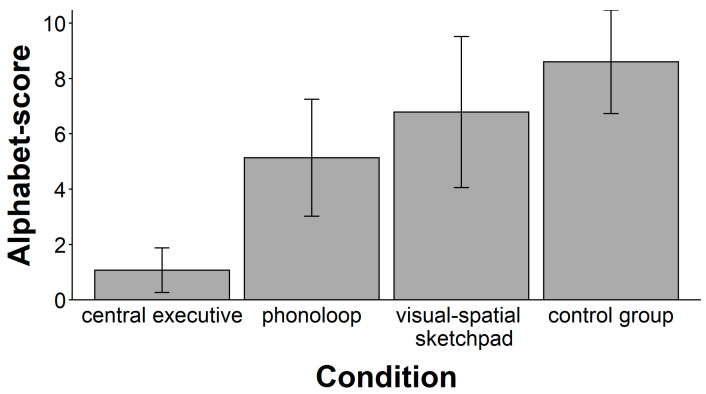
Mean performance in the alphabet task in the four different groups. Whiskers indicate 95% confidence intervals.

There was a significant main effect of the secondary task (see Figure 2), *F*(3, 54) = 11.77, *p* < .001, ω^2^ = 0.36. A follow-up with planned comparisons showed that the mean performance in the central executive condition was significantly lower than the mean performance in the other groups, *t*(56) = 5.08, *p* < .001, ω^2^ = 0.30. In contrast, the mean performance did not differ between the other three groups, *F*(2, 41) = 2.83, *p* = .071, ω^2^ = 0.07.

#### 3.2.3. Computation Span Task

**Figure 3:**
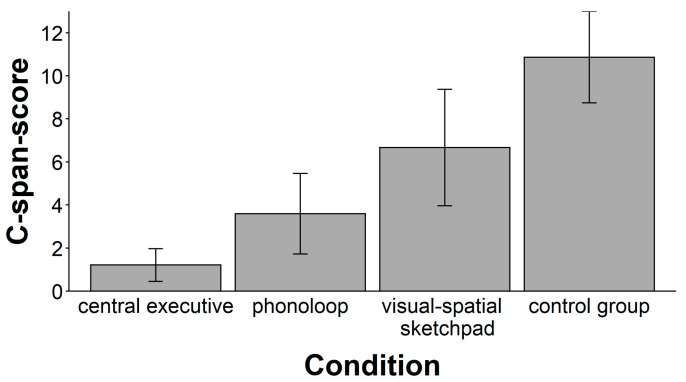
Mean performance in the computation span task in the four different groups. Whiskers indicate 95% confidence intervals.

There was a significant main effect of the secondary task (see Figure 3), *F*(3, 55) = 19.38, *p* < .001, ω^2^ = 0.48. A follow-up with planned comparisons showed that the mean performance in the central executive condition was significantly lower than the mean performance in the other groups, *t*(57) = 4.30, *p* < .001, ω^2^ = 0.23. There were also significant mean differences in performance between the other the groups, *F*(2, 42) = 12.00, *p* < .001, ω^2^ = 0.33. Post-hoc test with a Bonferroni–Holm adjustment of alpha levels for three comparisons (starting with α = 0.017) showed that mean performance in the control condition was higher than in the phonological loop condition (*t*(28) = 5.51, *p* < .001, ω^2^ = 0.49) and in the visuospatial sketchpad condition (*t*(28) = 2.62, *p* = .014, ω^2^ = 0.16), respectively. The mean performance in the phonological loop condition did not differ from the mean performance in the visuospatial sketchpad condition, *t*(28) = 2.00, *p* = .055, ω^2^ = 0.09.

#### 3.2.4. Letter Rotation Task

**Figure 4:**
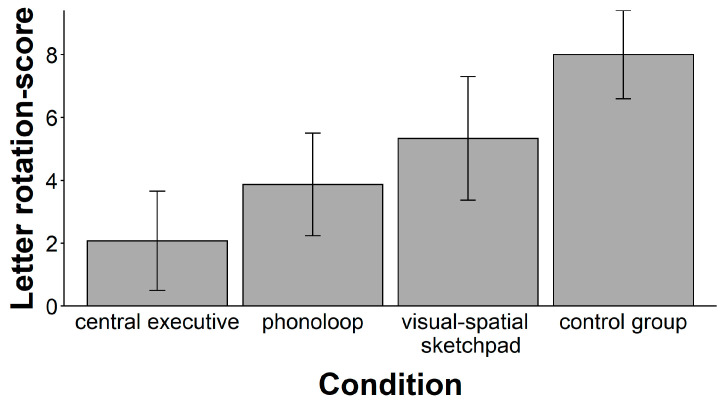
Mean performance in the letter rotation task in the four different groups. Whiskers indicate 95% confidence intervals.

Again, there was a significant main effect of the secondary task (see Figure 4), *F*(3, 55) = 10.37, *p* < .001, ω^2^ = 0.32. A follow-up with planned comparisons showed that the mean performance in the central executive condition was significantly lower than the mean performance in the other groups, *t*(57) = 3.64, *p* < .001, ω^2^ = 0.17. There were also significant mean differences in performance between the other the groups, *F*(2, 42) = 7.11, *p* = .002, ω^2^ = 0.21. Post-hoc test with a Bonferroni–Holm adjustment of alpha levels for three comparisons (starting with α = 0.017) showed that mean performance in the control condition was higher than in the phonological loop condition (*t*(28) = 4.12, *p* < .001, ω^2^ = 0.35) and in the visuospatial sketchpad condition (*t*(28) = 2.36, *p* = .025, ω^2^ = 0.13), respectively. The mean performance in the phonological loop condition did not differ from the mean performance in the visuospatial sketchpad condition, *t*(28) = 1.23, *p* = .229, ω^2^ = 0.02.

#### 3.2.5. Comparison of Study 1 vs. Study 2

In order to analyse whether the effect sizes of study 2 are significantly larger than the effect sizes of study 1, we merged the data of both studies. First, we z-standardized the APM total scores of study 1 across all four conditions to yield a generic performance measure. Second, we computed for each participant of study 2 the sum of the alphabet task, the computation span task, and the letter rotation task, and then z-standardized this sum score across all four conditions to yield a generic performance measure. We then subjected these performance scores to condition (4) by Study (2) ANOVA. As may be expected, there was a significant main effect of condition on performance, *F*(3, 111) = 19.93, *p* < .001, ω^2^ = 0.32. Most importantly, there was also a significant interaction of condition by study on performance, *F*(3, 111) = 3.11, *p* = .029, ω^2^ = 0.05. The effect of working memory load on performance was significantly larger when performance was measured with working memory tasks in study 2 than when it was measured with APM items in study 1.

### 3.3. Discussion

This study demonstrated that secondary tasks have a very strong impact on measures of working memory capacity. Loading the central executive with a random number generation task reliably impaired the performance in all three working memory tasks irrespective of their content. In addition, loading the phonological loop with a number repetition task or loading the visuospatial sketchpad with a key pressing task also deteriorated the performance in both the computation span task and the letter rotation task. Thus, all three working memory tasks engaged foremost a domain-general component of the working memory system, and some of those tasks also relied, to different degrees, on domain-specific components of working memory. This finding supports the validity of all three tasks for the measurement of “working memory capacity” as a domain-general factor (Oberauer et al. 2018), but it also renders some evidence to the notion that complex span tasks may be sensitive to the capacity of domain-specific systems, which has already been proposed by Baddeley (2007). In any case, this second study demonstrates how complex span tasks may be validated with an experimental approach, which complements the typical correlational approach in this field of research and adds further evidence for the validity of the alphabet task (Kyllonen and Christal 1990), the computation span task (Ackerman et al. 2002), and the letter rotation task (Miyake et al. 2001).

Vice versa, these findings also support the validity of the secondary tasks. In particular, the random number generation task presumably loads the central executive component of working memory and was therefore hypothesized to have a strong effect on the performance in complex working memory span tasks irrespective of their domain. The data clearly confirmed this prediction. Moreover, the other secondary tasks that presumably load specific slave systems of working memory showed more specific effects on the complex span tasks. Most importantly, the empirical effect sizes for secondary task effects on complex span performance were very large, reaching values of 32, 36, and 48% of explained variance. This result greatly supports the proposed validity of these secondary tasks (Baddeley 1986, 1997, 2007; Baddeley et al. 1998; Baddeley and Hitch 1974; Logie et al. 1990) and indeed shows that they are well suited to produce very large experimental effects.

## 4. General Discussion

The main result of the present study 1 is that an experimental manipulation of working memory load affected the performance in the APM. Loading the central executive with a secondary task reduced the number of correctly solved APM items, whereas loading the phonological loop or the visuospatial sketchpad had no deteriorating effect. This finding is consistent with the proposal that working memory capacity is intrinsically related to intelligence (Bühner et al. 2005; Colom et al. 2004; Engle et al. 1999; Kyllonen and Christal 1990). Previous research came to this conclusion by employing tests of working memory capacity and intelligence that have a certain overlap of item content (see the critique of Lohman (2005); Süß et al. (2002)), which may have resulted in an overestimation of the correlations between working memory capacity and intelligence. In study 1, we used a random number generation task, a number counting task, and a key pressing task as the independent variable and APM performance as the dependent variable. There is no obvious overlap of content, and thus the experimental effects cannot be attributed to the overlap of item or task contents.

Moreover, the findings of study 1 render further support for the proposition that it is the domain-general rather than domain-specific systems which sustain the performance in intelligence tests (Engle 2002). Most importantly, previous correlational studies came to this conclusion by employing tests of working memory capacity which supposedly measure a confound rather than a specific component of working memory. This measurement approach has been criticized by Baddeley (2007), who argued that none of these tests is a pure measure of any of these components. In study 1, we employed Baddeley’s (1986, 1997, 2007) own experimental research paradigm, which aims to load specific working memory components, and we also reached the conclusion that it is the central executive that contributes to performance in intelligence tests. In so far as this experimental approach is valid, the present finding of the starring role of the central executive must mitigate Baddeley’s (2007) concerns.

Another and perhaps even more important conclusion from the present study is that working memory capacity exerts a causal effect on the performance in a standard test of fluid intelligence. Previous studies aimed at such a conclusion with correlational designs, e.g., (Kyllonen and Christal 1990), have severe methodological shortcomings (Baddeley 2007; Shadish et al. 2002). The present study provides a more robust experimental demonstration of this causal effect. Loading working memory with different secondary tasks in a between-subject design manipulates the available capacity of working memory. Thus, the participants in a load condition have a smaller capacity available for solving the intelligence test items than the participants in a control condition. This experimental approach mimics naturally occurring individual differences in working memory capacity but may control for nuisance variables by randomization. Therefore, the causal nature of this effect is established by the present study.

The conclusion of the present study 1 is at odds with the proposal that “reasoning ability is little more than working-memory capacity” (Kyllonen and Christal 1990). In previous research, this conclusion has been reached in correlational studies by observing an association between working memory capacity and fluid intelligence that approaches unity. This conclusion may be criticized because there is a plethora of variables which exert effects on both working memory and intelligence test performance (such as motivation, speed and accuracy of information processing, neural efficiency, etc.; Baddeley (2007); Jensen (1998); Mackintosh (2011)). Whereas these criticisms are somewhat speculative insofar as these alternative explanations have not been rigorously tested in empirical research, the present study has manipulated working memory capacity in a randomized experiment and could show that this manipulation may explain 15 % of the variance in fluid intelligence test performance between individual participants. This rather low proportion of explained variance suggests that other factors than working memory capacity additionally contribute to individual differences in fluid intelligence.

Of course, when it is not the capacity of working memory that gives rise to the greater part of variance in fluid intelligence, which other factors may be at work? This pressing question may have found an answer with process overlap theory (POT; Kovacs and Conway (2016)), which explains the positive manifold by a domain-general set of executive functions. According to POT, working memory capacity and fluid intelligence share a substantial portion of their variance due to the executive function component of working memory tasks and not because of the storage capacity of these tasks (Kovacs and Conway 2020, p. 421). This explanation neatly fits to our result that loading the central executive deteriorates APM performance, whereas loading the phonological loop or the visuospatial sketchpad has no such effect. Although POT does not include a list of exactly what executive functions are in charge, Kovacs and Conway (2020) agreed that “Attentional control—also referred to as executive attention, cognitive control, executive control, inhibitory control, or executive functions—is an umbrella term that describes a wide variety of cognitive processes” (Schubert and Rey-Mermet 2019, p. 277). One subset of these processes may be the human capacity to build up and maintain the temporary bindings of elements that are stored in working memory (Oberauer et al. 2007, 2008), which may be measured with “relation-monitoring tasks” (Oberauer et al. 2008) or “relational integration tasks” (Chuderski 2014). It has been shown that these tasks are much better predictors of fluid intelligence than executive control tasks (Chuderski et al. 2012) and that relational integration tasks may predict fluid intelligence better than a variety of working memory tasks, including complex span tasks (Chuderski 2014). These findings suggest that the binding abilities of the cognitive system may be at the heart of domain-general processes that unfold the positive manifold.

One obvious objection to our conclusions is that they are only valid under the presumption that the secondary tasks of the present study yielded a sufficient loading of working memory. However, the present study 2 could demonstrate that loading the components of the working memory system exactly as in study 1 reduced the performance in three complex span tasks by about 40 %. Presuming the validity of these complex span tasks as a measure for “general working memory capacity” (Oberauer et al. 2018), this finding clearly underlines the validity of our experimental manipulation of working memory capacity. Because replacing the APM items by complex span tasks yielded a significant increase in the effect sizes by a factor of 2–3, we conclude that fluid intelligence is not (approximately) the same as working memory capacity. This conclusion is in line with Ackerman et al. (2005), who noted in their meta-analysis that the correlation between working memory capacity and intelligence is substantially less than unity and that these two are thus not isomorphic constructs. It is also in line with Oberauer et al. (2005), who noted in their meta-analysis that there is no theoretical reason to assert an isomorphism of working memory capacity and intelligence. Instead, they suggested that working memory capacity “should be regarded as an explanatory construct for intellectual abilities” (p. 61). The findings of our two studies render strong experimental evidence for this proposal.

### 4.1. Number of Solved Items vs. Reasoning Time

The aim of the present studies was an analysis of the effects of working memory capacity on fluid intelligence, the latter of which was measured as usual by scoring the numbers of correctly solved test items. Following the study of Rao and Baddeley (2013), however, we additionally used reasoning time as a dependent variable in some of the analyses. We found that the random number generation tasks diminished the APM performance as measured by the number of correctly solved items but did not significantly prolong reasoning time. Conversely, Rao and Baddeley (2013) reported a large effect of loading the central executive on SPM reasoning time but a considerably smaller effect on the number of correctly solved items. As it stands, loading working memory with a secondary task may have diminishing effects both on the number of correctly solved items and the speed of solving the items. Modern extensions of decay theories of working memory may explain this two-sided effect.

The component model of working memory of Baddeley and Hitch (1974; Baddeley 1986, 1997, 2007) already proposed that information in working memory decays over time if it is not refreshed and that the capacity of this memory system for the storage of information is limited. Therefore, performance in primary tasks diminishes when a secondary task is performed that impedes the refreshing of information and that uses a share of the system’s capacity. Moreover, a central executive component is involved with the control of processing and the allocation of limited attentional resources. Barrouillet et al. (2004; Barrouillet and Camos 2007) elaborated on this idea and suggested a time-based resource-sharing (TBRS) model of working memory. They proposed that working memory is a quickly switching, serial device that focuses executive attention on a single memory trace at a time to restore its activation (i.e., accessibility). This process is termed “attentional refreshing”, and it counteracts the continuous temporal decay of items in working memory. Most importantly, the TBRS model presumes that processing and maintenance of information rely on the same attention resource, which is limited.

Given a constant speed of information processing (say, in bits/s), a secondary task will need some time per second for its processing, thus less time per second is available for the primary task. Moreover, switching costs for switching between the primary task and the secondary task may emerge. This must result in a prolonged time span that is required for conducting all necessary processing steps to generate a solution for the primary task. In the same vein, this leaves less time for refreshing the memory traces for the primary task, thus some information decays, and tasks that require the maintenance of more information (i.e., more difficult matrix items) cannot be solved.

In the case of SPM items that are of rather low difficulty, a person may still solve an item even when his or her central executive is loaded with a secondary task, but the price is a deceleration of the solving process. In case of the APM items that are of greater difficulty, however, even a deceleration of the solving process cannot compensate for the loss of capacity due to loading the central executive with a secondary task, thus the person may not be able to solve the item and may reach this insight rather quickly and give up. Thus, the TBRS model accounts for the finding that loading the central executive with a secondary task may both reduce the number of correctly solved items and increase reasoning time, while the salient effect may depend on the difficulty of the primary task. Future research could address the utility of this model for a better explanation of the association between working memory and intelligence.

### 4.2. Limitations

The main conclusions of the two experiments are that there are causal effects of working memory capacity on fluid intelligence, and that working memory capacity and fluid intelligence are not isomorphic, i.e., that intelligence is much more than working memory capacity. As with any experiment, these conclusions rest on the presumed validity of the independent and the dependent measures.

First, we used items of the APM to measure “fluid intelligence” in study 1. The decision to use this test was based on findings from factor-analytic research and nonmetric scaling studies, which demonstrated that this test has a maximum loading on fluid intelligence and that it is at the core of the cognitive ability space, and because there is a general agreement in the literature that the APM may serve as a good measure of fluid intelligence (Carroll 1993; Carpenter et al. 1990; Chooi and Thompson 2012; Colom et al. 2015; Jaeggi et al. 2008; Mackintosh 2011).

Second, we used complex span tasks to measure working memory capacity in study 2. These tasks have been used and validated in previous research, see (Ackerman et al. 2002; Kyllonen and Christal 1990; Miyake et al. 2001), and it is a benchmark finding that complex span tasks show a positive manifold, which points to a common factor that has been termed “general working memory capacity” (Oberauer et al. 2018). It is obvious that many different basic cognitive operations are involved while the participants work on these tasks, and therefore the capacity measures reflect a “syndrome” of different processes in working memory rather than a specific “symptom” of working memory functioning. Study 2 supports this conclusion because we combined the *same* secondary tasks with *different* complex span tasks from the verbal, numerical, or spatial domain and found three *different* profiles of impairment due to a strain of the cognitive system by the secondary tasks (see Figure 2, Figure 3 and Figure 4). Thus, although all three complex span tasks measure the capacity of “working memory”, they do not appear to be isomorphic.

Third, we used secondary tasks to reduce the working memory capacity of our participants. These tasks were developed and validated by Baddeley and colleagues (Baddeley 1986, 1997, 2007; Baddeley et al. 1998; Baddeley and Hitch 1974; Logie et al. 1990) and these tasks have been repeatedly shown to impair the performance in a variety of primary tasks. Moreover, Baddeley and colleagues have developed a theoretical account that may explain these findings in terms of the functioning of “working memory”. Nonetheless, it is not clear what exactly happens in these tasks on the level of basic cognitive operations. For example, the random number generation task that was used to manipulate the functions of the central executive certainly engages a variety of cognitive operations, such as activating a set of numbers that can be used, binding of the numbers to the positions in the sequence that is produced, binding of the numbers to their frequency in the sequence, comparing the frequencies of numbers in the sequence, selecting numbers with low frequencies, updating the bindings of number position and number frequency, and so on. Thus, this task manipulates a “syndrome” rather than a specific “symptom”. Today, there is a consensus that “working memory” is a complex construct that is related to a plethora of empirical findings and that there is no general theory to explain it (Oberauer et al. 2018). The findings of study 2 clearly suggest that these secondary tasks experimentally manipulate something that may be termed “working memory capacity”. Thus, we are confident that the secondary tasks we used offer a valid method to manipulate the efficiency of working memory on a global level, no matter which specific sub-processes of working memory are involved.

A final limitation stems from the two samples of participants that provided the data of the present work. Both samples consisted of university students in their early twenties, and it cannot be taken for granted that the cognitive architecture of this age group is even approximately representative for the whole life span. For example, Demetriou et al. (2018) suggested that intelligence is a function of a variety of processes such as attention control, flexibility, working memory, cognizance, and inference. In an extensive review of the literature, they showed that in the first two decades of life “the contribution of attention control and flexibility diminishes but the contribution of working memory, cognizance, and inference increases” (Demetriou et al. 2018, p. 20). Thus, it may be possible that the functional role of working memory for fluid intelligence does change across the life span and that the findings of the present work are limited to young adults.

Taken together, we used well-established tasks and measures as independent and dependent variables in both experiments, and the findings of our two experiments fit well and in a predictable way into a nomothetic network of working memory capacity and fluid intelligence. This does not mitigate the need for more research, which elucidates the basic cognitive processes of secondary tasks or of complex span tasks in different age groups. This kind of enquiry, however, was neither the aim nor within the scope of the present work.

## 5. Conclusions

Using Baddeley’s (1986, 1997, 2007) multicomponent theory of working memory as a theoretical framework, the present study provided evidence that the available capacity of the central executive may have a causal effect on the performance in a test of fluid intelligence, whereas the capacity of the phonological loop or the visuospatial sketchpad were not related to test performance. Only a total of 15% of the variance in the intelligence test performance could be explained by the manipulation of working memory capacity, whereas the very same manipulation exerted an experimental effect of 2–3 times this size when the dependent variable was replaced with complex working memory span tasks. From this finding, we conclude that working memory capacity is not the only cognitive factor that determines fluid intelligence, but that there must be other factors contributing to the variance in intelligence, such as speed of information processing, attention, memory access, and transfer of information into long-term memory, just to name some of them (Mackintosh 2011; Schweizer 2005).

## Data Availability

The data supporting reported results can be found in the Open Science Framework repository: https://osf.io/hdcvj/.

## References

[B1-jintelligence-11-00070] Ackerman Phillip L., Beier Margaret E., Boyle Mary O. (2002). Individual differences in working memory within a nomological network of cognitive and perceptual speed abilities. Journal of Experimental Psychology: General.

[B2-jintelligence-11-00070] Ackerman Phillip L., Beier Margaret E., Boyle Mary O. (2005). Working memory and intelligence: The same or different constructs?. Psychological Bulletin.

[B3-jintelligence-11-00070] Baddeley Allan D. (1986). Working Memory.

[B4-jintelligence-11-00070] Baddeley Allan D. (1997). Human Memory. Theory and Practice.

[B5-jintelligence-11-00070] Baddeley Allan D. (2007). Working Memory, Thought, and Action.

[B6-jintelligence-11-00070] Baddeley Allan D., Hitch Graham (1974). Working memory. The Psychology of Learning and Motivation.

[B7-jintelligence-11-00070] Baddeley Allan D., Emslie Hazel, Kolodny Jonathan, Duncan John (1998). Random generation and the executive control of working memory. Quarterly Journal of Experimental Psychology Section A.

[B8-jintelligence-11-00070] Baddeley Allan D., Lewis Vivien L., Vallar Giuseppe (1984). Exploring the articulatory loop. Quarterly Journal of Experimental Psychology.

[B9-jintelligence-11-00070] Barrouillet Pierre, Camos Valerie, Osaka Naoyuki, Logie Robert H., D’Esposito Mark (2007). The time-based resource-sharing model of working memory. The Cognitive Neuroscience of Working Memory.

[B10-jintelligence-11-00070] Barrouillet Pierre, Bernardin Sophie, Camos Valerie (2004). Time Constraints and Resource Sharing in Adults’ Working Memory Spans. Journal of Experimental Psychology: General.

[B11-jintelligence-11-00070] Bühner Markus, Krumm Stefan, Pick Marion (2005). Reasoning=working memory≠attention. Intelligence.

[B12-jintelligence-11-00070] Carpenter Patricia. A., Just Marcel A., Shell Peter (1990). What one intelligence test measures: A theoretical account of the processing in the Raven progressive matrices test. Psychological Review.

[B13-jintelligence-11-00070] Carroll John B. (1993). Human Cognitive Abilities. A Survey of Factor-Analytic Studies.

[B14-jintelligence-11-00070] Cattell Raymond B. (1973). Measuring Intelligence with the Culture Fair Tests.

[B15-jintelligence-11-00070] Cattell Raymond B., Weiß Rudolf H. (1971). Grundintelligenztest CFT 3—Skala 3.

[B16-jintelligence-11-00070] Chooi Weng-Tink, Thompson Lee A. (2012). Working memory training does not improve intelligence in healthy young adults. Intelligence.

[B17-jintelligence-11-00070] Chuderski Adam (2014). The relational integration task explains fluid reasoning above and beyond other working memory tasks. Memory & Cognition.

[B18-jintelligence-11-00070] Chuderski Adam, Taraday Maciej, Necka Edward, Smolen Tomasz (2012). Storage capacity explains fluid intelligence while executive control does not. Intelligence.

[B19-jintelligence-11-00070] Cohen Jacob (1988). Statistical Power Analysis for the Behavioral Sciences.

[B20-jintelligence-11-00070] Colom Roberto, Rebollo Irene, Palacios Antonio, Juan-Espinosa Manuel, Kyllonen Patrick C. (2004). Working memory is (almost) perfectly predicted by g. Intelligence.

[B21-jintelligence-11-00070] Colom Roberto, Privado Jesús, García Luis F., Estrada Eduardo, Cuevas Lara, Shih Pei C. (2015). Fluid intelligence and working memory capacity: Is the time of working on intelligence problems relevant for explaining their large relationship?. Personality and Individual Differences.

[B22-jintelligence-11-00070] Conway Andrew R. A., Kane Michael J., Bunting Michael F., Hambrick D. Zach, Wilhelm Oliver, Engle Randall W. (2005). Working memory span tasks: A review and a user’s guide. Psychonomic Bulletin and Review.

[B23-jintelligence-11-00070] Conway Andrew R. A., Cowan Nelson, Bunting Michael F., Therriault David J., Minkoff Scott R. B. (2004). A latent variable analysis of working memory capacity, short term memory capacity, processing speed, and general fluid intelligence. Intelligence.

[B24-jintelligence-11-00070] Cowan Nelson, Miyake Akira, Shah Priti (1999). An embedded-processes model of working memory. Models of Working Memory: Mechanisms of Active Maintenance and Executive Control.

[B25-jintelligence-11-00070] Cowan Nelson (2017). The many faces of working memory and short-term storage. Psychonomic Bulletin & Review.

[B26-jintelligence-11-00070] Cronbach Lee. J. (1957). The two disciplines of scientific psychology. American Psychologist.

[B27-jintelligence-11-00070] De Simoni Carla, von Bastian Claudia C. (2018). Working memory updating and binding training: Bayesian evidence supporting the absence of transfer. Journal of Experimental Psychology: General.

[B28-jintelligence-11-00070] Demetriou Andreas, Makris Nikolas, Spanoudis George, Kazi Smaragda, Shayer Michael, Kazali Elena (2018). Mapping the dimensions of general intelligence: An integrated differential-developmental theory. Human Development.

[B29-jintelligence-11-00070] Engle Randall W. (2002). Working memory capacity as executive attention. Current Directions in Psychological Science.

[B30-jintelligence-11-00070] Engle Randall W., Tuholski Stephen W., Laughlin James E., Conway Andrew R. A. (1999). Working memory, short-term memory, and general fluid intelligence: A latent-variable approach. Journal of Experimental Psychology: General.

[B31-jintelligence-11-00070] Erdfelder Edgar, Faul Franz, Buchner Axel (1996). GPOWER: A general power analysis program. Behavior Research Methods, Instruments, & Computers.

[B32-jintelligence-11-00070] Farmer Eric. W., Berman Jonathan V., Fletcher Yvonne L. (1986). Evidence for a visuospatial scratch pad in working memory. Quarterly Journal of Experimental Psychology.

[B33-jintelligence-11-00070] Farrell Simon, Lewandowsky Stephan (2002). An endogenous distributed model of ordering in serial recall. Psychonomic Bulletin & Review.

[B34-jintelligence-11-00070] Gilhooly Kenneth J., Logie Robert H., Wetherick Norman E., Wynn Valerie E. (1993). Working memory and strategies in syllogistic-reasoning tasks. Memory & Cognition.

[B35-jintelligence-11-00070] Hays William L. (1994). Statistics.

[B36-jintelligence-11-00070] Hossiep Rüdiger, Turck Daniela, Hasella Michele (1999). Bochumer Matrizentest: BOMAT–Advanced–Short Version.

[B37-jintelligence-11-00070] Jaeggi Susanne M., Buschkuehl Martin, Jonides John, Perrig Walter J. (2008). Improving fluid intelligence with training on working memory. Proceedings of the National Academy of Sciences of the United States of America.

[B38-jintelligence-11-00070] Jensen Arthur R. (1981). Straight Talk about Mental Tests.

[B39-jintelligence-11-00070] Jensen Arthur R. (1998). The g Factor. The Science of Mental Ability.

[B40-jintelligence-11-00070] Kane Michael J., Hambrick David Z., Conway Andrew R. A. (2005). Working memory capacity and fluid intelligence are strongly related constructs: Comment on Ackerman, Beier, and Boyle. Psychological Bulletin.

[B41-jintelligence-11-00070] Kane Michael J., Hambrick David Z., Tuholski Stephen W., Wilhelm Oliver, Payne Tabitha W., Engle Randall W. (2004). The generality of working memory capacity: A latent-variable approach to verbal and visuospatial memory span and reasoning. Journal of Experimental Psychology: General.

[B42-jintelligence-11-00070] Klauer Karl C., Stegmaier Ralf, Meiser Thorsten T. (1997). Working memory involvement in propositional and spatial reasoning. Thinking and Reasoning.

[B43-jintelligence-11-00070] Kovacs Kristof, Conway Andrew R. A. (2016). Process overlap theory: A unified account of the general factor of intelligence. Psychological Inquiry.

[B44-jintelligence-11-00070] Kovacs Kristof, Conway Andrew R. A. (2020). Process overlap theory, executive functions, and the interpretation of cognitive test scores: Reply to commentaries. Journal of Applied Research in Memory and Cognition.

[B45-jintelligence-11-00070] Kyllonen Patrick. C., Sternberg Robert. J., Gigorenko Elena. L. (2002). g: Knowledge, speed, strategies, or working memory capacity? A systems perspective. The general factor of intelligence: How general is it?.

[B46-jintelligence-11-00070] Kyllonen Patrick. C., Christal Raymond E. (1990). Reasoning ability is (little more) than working-memory capacity?!. Intelligence.

[B47-jintelligence-11-00070] Logie Robert H., Baddeley Alan D., Mané Amir, Donchin Emanuel, Sheptak Russell (1989). Working memory in the acquisition of complex cognitive skills. Acta Psychologica.

[B48-jintelligence-11-00070] Logie Robert H., Zucco Gesualdo, Baddeley Alan D. (1990). Interference with visual short-term memory. Acta Psychologica.

[B49-jintelligence-11-00070] Lohman David. F., Sternberg Robert J., Pretz Jean E. (2005). Reasoning abilities. Cognition and Intelligence.

[B50-jintelligence-11-00070] Mackintosh Nicholas (2011). IQ and Human Intelligence.

[B51-jintelligence-11-00070] Melby-Lervac Monica, Redick Thomas S., Hulme Charles (2016). Working memory training does not improve performance on measures of intelligence or other measures of “far transfer”: Evidence from a meta-analytic review. Psychological Science.

[B52-jintelligence-11-00070] Miyake Akira, Friedman Naomi P., Rettinger David A., Sha Priti, Hegarty Mary (2001). How are visuospatial working memory, executive functioning, and spatial analysis related? A latent-variable analysis. Journal of Experimental Psychology: General.

[B53-jintelligence-11-00070] Oberauer Klaus, Süß Hans-Martin, Wilhelm Oliver, Sander Nicolas, Conway Andrew R. A., Jarrold Christopher, Kane Michael J., Miyake Akira, Towse John N. (2007). Individual differences in working memory capacity and reasoning ability. Variation in Working Memory.

[B54-jintelligence-11-00070] Oberauer Klaus, Süß Hans-Martin, Wilhelm Oliver, Wittmann Werner W. (2008). Which working memory functions predict intelligence?. Intelligence.

[B55-jintelligence-11-00070] Oberauer Klaus, Schulze Ralf, Wilhelm Oliver, Süß Heinz-Martin (2005). Working memory and intelligence—Their correlation and their relation: Comment on Ackerman, Beier, and Boyle. Psychological Bulletin.

[B56-jintelligence-11-00070] Oberauer Klaus, Lewandowsky Stephan, Awh Edward, Brown Gordon D. A., Conway Andrew, Cowan Nelson, Donkin Christopher, Farrell Simon, Hitch Graham J., Hurlstone Mark J. (2018). Benchmarks for models of short-term and working memory. Psychological Bulletin.

[B57-jintelligence-11-00070] Oberauer Klaus (2019). Working memory capacity limits memory for bindings. Journal of Cognition.

[B58-jintelligence-11-00070] Popov Vencislav, Reder Lynne M. (2020). Frequency effects on memory: A resource-limited theory. Psychological Review.

[B59-jintelligence-11-00070] Rao K. Venkata, Baddeley Alan (2013). Raven’s matrices and working memory: A dual-task approach. Quarterly Journal of Experimental Psychology.

[B60-jintelligence-11-00070] Raven John C., Court John H., Raven John E. (1977a). Raven’s Progressive Matrices and Vocabulary Scales.

[B61-jintelligence-11-00070] Raven John C., Court John H., Raven John E. (1977b). Standard Progressive Matrices.

[B62-jintelligence-11-00070] Redick Thomas S., Shipstead Zach, Harrison Tyler L., Hicks Kenny L., Fried David E., Hambrick David Z., Kane Michael J., Engle Randall W. (2013). No evidence of intelligence improvement after working memory training: A randomized, placebo-controlled study. Journal of Experimental Psychology: General.

[B63-jintelligence-11-00070] Schmiedek Florian, Lövdén Martin, Lindenberger Ulman (2010). Hundred days of cognitive training enhance broad cognitive abilities in adulthood: Findings from the COGITO study. Frontiers in Aging Neuroscience.

[B64-jintelligence-11-00070] Schmiedek Florian, Lövdén Martin, Lindenberger Ulman (2014). Younger adults show long-term effects of cognitive training on broad cognitive abilities over 2 years. Developmental Psychology.

[B65-jintelligence-11-00070] Schubert Anna-Lena, Rey-Mermet Alodie (2019). Does process overlap theory replaces the issues of general intelligence with the issues of attentional control?. Journal of Applied Research in Memory and Cognition.

[B66-jintelligence-11-00070] Schweizer Karl (2005). An overview of research into the cognitive basis of intelligence. Journal of Individual Differences.

[B67-jintelligence-11-00070] Shadish William. R., Cook Thomas D., Campbell Donald T. (2002). Experimental and Quasi-Experimental Designs for Generalized Causal Inference.

[B68-jintelligence-11-00070] Steyvers Mark, Schafer Robert J. (2020). Inferring latent learning factors in large-scale cognitive training data. Nature Human Behaviour.

[B69-jintelligence-11-00070] Süß Heinz-Martin, Oberauer Klaus, Wittmann Werner W., Wilhelm Oliver, Schulze Ralf (2002). Working memory capacity explains reasoning ability—And a little bit more. Intelligence.

[B70-jintelligence-11-00070] Unsworth Nash, Engle Randall W. (2007). The nature of individual differences in working memory capacity: Active maintenance in primary memory and controlled search from secondary memory. Psychological Review.

[B71-jintelligence-11-00070] Wechsler David (1939). Wechsel-Bellvue Intelligence Scale.

[B72-jintelligence-11-00070] Wechsler David (1955). Wechsler Adult Intelligence Scale.

[B73-jintelligence-11-00070] Zhang Weiwei, Luck Steven J. (2008). Discrete fixed-resolution representations in visual working memory. Nature.

[B74-jintelligence-11-00070] Zhu Jianjun, Weiss Larry, Flanagan Dawn P., Harrison Patti L. (2005). The Wechsler scales. Contemporary Intellectual Assessment.

